# Clinical Efficacy of Topical CoQ10 and Vitamin-E Eye-drop in Retinopathy of Prematurity

**Published:** 2019-10-01

**Authors:** Muberra Akdogan, Onur Polat

**Affiliations:** 1 Afyonkarahisar Health Sciences University, Faculty of Medicine, Department of Ophthalmology, Afyonkarahisar, Turkey; 2 Dunyagoz Hospital, Ophthalmology Clinic, Bursa, Turkey

**Keywords:** Oxidative Stress, Retinopathy of Prematurity, Endothelial Growth Factors, Topical CoQ10, Vitamin-E Eye-drop

## Abstract

Treatment strategy for retinopathy of prematurity (ROP) includes anti-vascular endothelial growth factor (anti-VEGF) and/or laser therapy. The aim of this study was to investigate the clinical effects of topical Coqun® eye drop (CoQ10 and Vitamin-E) on the progression and treatment of ROP. One hundred and ten infants with type 1 ROP who received Coqun® (Coqun group) and 131 infants with type 1 ROP who did not receive Coqun® (control group) were included in this retrospective analysis. All patients were follow-up until retinal vascular maturation was complete. Intravitreal bevacizumab (IVB) injection or laser photocoagulation (LPC) were apply if needed. Treatment frequency, treatment response and mean follow-up time were compare. The number of IVB was similar between the groups, but infants in the Coqun group underwent significantly fewer LPC procedure than those in the control group (P = 0.022). The mean follow-up time was significantly shorter in infants receiving Coqun® in stage 1 ROP (P = 0.017) and similar in stages 2-4 ROP and aggressive posterior retinopathy of prematurity (APROP). The number of LPC procedure was fewer in the Coqun group in APROP (P = 0.043). These results indicate that faster retinal vascular maturation in infants with low-grade ROP and lower number of treatments with APROP could be achieve with Coqun® therapy.

## INTRODUCTION

Retinopathy of prematurity (ROP) is an oxidative-stress-related disease of incompletely vascularized retina of the low birth weight and premature infants. Prompt diagnosis and early management is critical to prevent blindness [1, 2]. Recent technical advancements in the neonatal intensive care units have markedly improved the survival rates of low birth weight infants and hence increased the incidence of ROP worldwide [3]. Retinopathy of prematurity is a multifactorial disease and low birth weight, low birth week and oxygen dysregulation are among the most significant risk factors [4, 5]. If ROP is left untreated, it may lead to vision-threatening complications such as ectopic macula, retinal detachment, angle-closure glaucoma or pupillary block. Even though, it may result in long-term sequelae such as myopia, strabismus or amblyopia [2, 6].

The association between hyperoxia and ROP has long been known. Premature infants are at increased risk of oxidative stress due to immature antioxidant systems [7]. Oxidative stress induced free oxygen radicals contribute to mitochondrial and electron transport chain (ETC) dysfunction and cause ischemic damage which is one of the main mechanisms underlying ROP development [8]. Coenzyme Q10 (CQ10, Ubiquinone), an important cofactor in ETC, is a potent antioxidant shown to have protective effects in experimental animal models of retinopathy and in neurodegenerative disorders such as Alzheimer's and Parkinson's diseases [9-12].

We have recently investigated the efficacy of Coqun® (Visufarma, Rome), which contains CQ10 and Vitamin E on the thiol-disulphide homeostasis, a novel oxidative stress marker in the ROP disease course [13]. To the best of our knowledge, there is no study on the clinical effects of CQ10 in premature infants. In this study, we sought to evaluate the effects of Q10/Vitamin E combination coenzyme on the progression and treatment of ROP.

## METHODS

We retrospectively reviewed the records of premature infants admitted to ROP department of ophthalmology clinic between February 2016 and February 2017 in Bursa Yuksek Ihtisas Health Science University, Bursa, Turkey. Infants with a ROP diagnosis at any stage who received Coqun® were included in the study. The protocol was approved by the Ethics Committee of HSU Bursa Yuksek Ihtisas Training and Research Hospital (2011-KAEK 2018/08.06). Moreover, the study adhered to the tenets of the Declaration of Helsinki.

All infants born below 37 weeks of gestational age (GA) were examined. Those with a ROP diagnosis whose parents’ consent was obtained to receive Coqun® were assigned into the Coqun group and those who had a ROP diagnosis but did not receive Coqun® were assigned to the control group. The first ROP examination of infants with a GA of ≤ 27 weeks was performed at 30-31 weeks and of those with a GA of ≥ 28 weeks was done 4 weeks after birth. Infants who already had a ROP diagnosis and/or were already receiving ROP treatment, those with stage 5 ROP, anterior segment disorders or a congenital anomaly, systemic complications such as sepsis, acidosis, intraventricular hemorrhage, an indistinguishable fundus appearance and those who continued their treatment in another center were excluded.

Oral feeding was discontinued one hour before ophthalmic examination and 0.5% tropicamide (Tropamid® Bilim Pharmaceuticals, Turkey) and 2.5% phenylephrine (Mydfrine®, Alcon, USA) eye drops were administered to achieve and maintain pupil dilation. Thereafter, a sterile lid speculum was placed under topical anesthesia using proparacaine hydrochloride (Alcaine®, Alcon, USA). The anterior segment, optic disc and macula were evaluated using a +28 diopter lens and indirect binocular ophthalmoscopy (Omega 500, Heine, Germany). Peripheral retinal examination was extended to the ora serrata using a scleral depressor. All retinal images were recorded with an Archimed Medical video archiving system (Pronova, Turkey). The use of Coqun® was recommended twice a day one drop for infants with a ROP diagnosis of any stage until retinal vascularization reached the ora serrata.

We used the Early Treatment of Retinopathy of Prematurity (ETROP) criteria to make a treatment decision and the International Classification of Retinopathy of Prematurity (ICROP) categorization to classify ROP [14, 15]. Accordingly, infants with stages 0-1 ROP were classified as Group 1, stages 1-2 ROP as Group 2, infants with at least 2 clock hours of stage 3 ROP or stage 4 ROP as Group 3 and those with aggressive posterior retinopathy of prematurity (APROP) (it is characteristically in a more posterior location with rapid progression of stage 1 to 5 and poor prognosis) [16] were classified as Group 4. 

Treatment was consider for eyes with zone I stage 1-2 plus disease, zone II stage 2-3 plus disease and for infants with APROP. Based on the BEAT-ROP study criteria, we used Intravitreal bevacizumab (IVB) (Altuzan®, Roche, Switzerland) 0.16-0.32 mg (very low-low dose) injection as the first-line treatment [17, 18]. IVB was performed in the operating room under local anesthesia (0.5% propacaine hydrochloride drop (Alcaine®, Alcon, USA) using a 32-gauge needle placed into the vitreous cavity 0.5-1 mm posterior to the limbus. The anti-VEGF dose in groups 1, 2 and 3 was 0.16 mg and in group 4 (AP-ROP) 0.32 mg. Infants who did not respond to anti-VEGF treatment, i.e. if ROP did not regress and/or progress within two weeks after the treatment, were treated with diode laser photocoagulation (LPC).

Laser photocoagulation was performed in the operating room under general anesthesia using an 810-nanometer (nm) diode laser (Iridex; Oculight SL, USA) and by leaving a half-shoot space (150–200 milliwatt [mW] of power for 0.2 second [s]). Infants who were treated with LPC received netilmicin sulfate 3% (Netira®, Teka, Turkey) and dexamethasone (Maxidex, Alcon, USA) eye drops every 6 hours one drop for one week. Those who were treated with anti-VEGF injection received netilmicin eye drops every six hours one drop and betaxolol hydrochloride eye drop (Betoptic-s®, Alcon, USA) once-a-day for one week. Follow-up was conducted at postoperative days 1, 3 and 7. The efficiency of anti-VEGF treatment was assessed one week after the procedure and of LPC treatment at postoperative weeks 2 to 5. The frequency of follow-up varied depending on the physical examination findings.

Infants who received anti-VEGF treatment, those who were treated with LPC as well as those who did not receive any treatment were followed-up until retinal vascularization was complete. Similarly, Coqun®, twice-a-day one drop, was continued until retinal vascularization was complete.

Gestational age, birth weight (BW), length of stay in the incubator, follow-up time, anterior segment and fundus examination findings, localization and stage of ROP and presence of a plus disease as well as the treatment procedure were recorded. We compared ROP stages, treatment frequency, treatment response and mean follow-up time between the two groups.

Statistical analysis was performed using SPSS Version 18.0 (SPSS, Inc., IL, USA). We used Kolmogorov-Smirnov test to assess normality of distribution. For normally distributed data, comparison between the two groups was performed using independent samples t-test, and for abnormally distributed data the Mann Whitney U test. Statistical significance was set at a P-value of < 0.05 and confidence interval of 95%.

## RESULTS

A total of 482 eyes of 241 infants were included in the study. Of these 110 (58 females, 52 males) were assigned in the Coqun® group and 131 (61 females, 70 males) in the control group. The mean ± standard deviation (SD) of GA in the Coqun® and control groups were 29.33 ± 2.87 (range, 23-35) weeks and 29.14 ± 2.89 (range, 23-34) weeks, respectively. Groups were well matched regarding GA and gender (P = 0.43 and P = 0.36). The average birth weights, the mean follow-up times and the mean length of stay in the incubator times were similar between the two groups (P = 0.62, P = 0.13 and P = 0.73, respectively). 

**Table 1:** Demographics of the Patients in Coqun and Control Groups

	Coqun®	Control	P-value
Sex			0.36
**Male; n**	52	70	
**Female; n**	58	61	
GA, weeks; Mean ± SD	29.33 ± 2.87	29.14 ± 2.89	0.43
BW, g; Mean ± SD	1255.41 ± 445.55	1291.84 ± 475.45	0.62
Incubator time, days; Mean ± SD	49.7 ± 32.1	51.3 ± 32.2	0.73
Follow-up time, weeks; Mean ± SD	48.82 ± 7.31	50.59 ± 7.65	0.13
Anti-VEGF injection; n (%)	26 (23.6)	30 (22.9)	0.65
Laser photocoagulation; n (%)	12 (10.9)	32 (24.4)	**0.022**

Abbreviations: SD: standard deviation; GA: Gestational age; BW: Birth weight; n: number; %: percentage; g: gram; Anti-VEGF: anti-vascular endothelial growth factor; Coqun: topical Coqun® eye drop (CoQ10 and Vitamin-E). P < 0.05: Statistically difference in laser photocoagulation numbers between the groups.

**Table 2:** Subgroup Analyses in the Coqun® and Control Groups

	Coqun® (+)	Control	P-value
Group 1 (n = 42/45)			
**GA at birth (weeks)**	30.62 ± 2.56	30.53 ± 2.56	0.87
**Birth weight (g)**	1487.1 ± 441.3	1512.8 ± 540.1	0.93
**Follow-up time (weeks)**	44.95 ± 5.71	47.26 ± 5.67	**0.017**
Group 2 (n = 35/38)			
**GA at birth (weeks)**	29.14 ± 2.55	29.89 ± 2.05	0.16
**Birth weight (g)**	1247.5 ± 420.1	1303.8 ± 377.7	0.37
**Follow-up time (weeks)**	50.85 ± 7.09	52.02 ± 8.58	0.66
Group 3 (n = 15/19)			
**GA at birth (weeks)**	28.26 ± 2.73	29.84 ± 2.85	0.13
**Birth weight (g)**	1112.3 ± 419.8	1270.3 ± 462.5	0.29
**Follow-up time (weeks)**	48.50 ± 8.88	53.76 ± 8.47	0.31
Group 4 (n = 18/29)			
**GA at birth (weeks)**	26.44 ± 2.17	26.41 ± 2.16	0.96
**Birth weight (g)**	862.2 ± 233.3	954.9 ± 269.2	0.25
**Follow-up time (weeks)**	54.72 ± 6.24	53.53 ± 6.73	0.67

Data in table are presented as Mean ± SD.

Abbreviations: SD: standard deviation; n: number; GA: Gestational age; g: gram; Coqun: topical Coqun® eye drop (CoQ10 and Vitamin-E). P < 0.05: Statistically difference in follow-up time between the groups.

**Figure 1:**
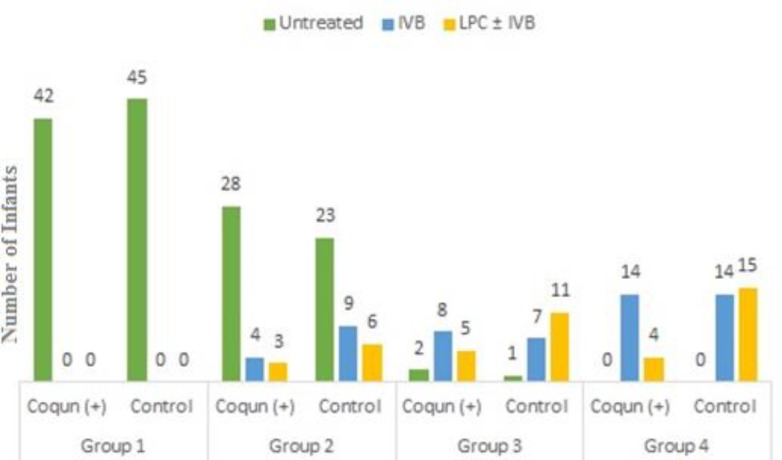
The number of treated and untreated infants in the subgroup analysis

Group 1: infants with stages 0-1 ROP; Group 2: infants with stages 1-2 ROP; Group 3: infants with at least 2 clock hours of stage 3 ROP or stage 4 ROP; Group 4: infants with aggressive posterior retinopathy of prematurity (APROP). IVB: Intravitreal bevacizumab; LPC: Laser photocoagulation; Coqun: topical Coqun® eye drop (CoQ10 and Vitamin-E). The culumns represent the number of infants.

Demographic data is shown in Table 1. Subgroup analysis showed no significant difference in mean GA and mean BW between any of the groups (Table 2). The mean follow-up time was statistically shorter in infants receiving drug treatment in Group 1 (P = 0.017) but it was not statistically significant between other groups (P = 0.66, P = 0.31, P = 0.67) (Table 2).

The number of untreated infants in the Coqun® and control groups were 72 (65.5%) and 69 (52.7%), respectively. The number of infants who received an intravitreal injection in the Coqun® and control groups were 26 (23.6%) and 30 (22.9%), respectively. Infants in the control group underwent significantly more Diode LPC procedures than those in the Coqun® group [32 (24.4%) versus 12 (10.9%); P = 0.022]. 

The number of treated and untreated infants in the subgroup analysis are presented in Figure 1. Accordingly, in Group 1, retinal maturation was complete in all infants in the Coqun® and control groups without any IVB or LPC treatment. In Group 2, the number of infants who received any means of treatment was 7 in the Coqun® and 15 in the control groups (P = 0.19) and in Group 3, 13 in the Coqun® and 18 in the control groups (P = 0.33). In Group 4, while the number of infants treated with IVB was equal in the both groups, significantly more infants underwent LPC in the control group than the Coqun® one (15 versus 4; P = 0.043).

## DISCUSSION

In the current study, we found that the use of Q10/Vitamin E combination coenzyme provided faster retinal maturation with fewer treatments in infants with ROP. 

The pathophysiology of ROP is characterized by a two-phase process. In phase-1, the outer retinal layers are not yet developed, thus retinal metabolic activity is low. During the first weeks of birth, the infant is exposed to an increased oxygen pressure due to a transition from intrauterine to extrauterine environment. Given the intrauterine physiologic hypoxic status, this transition causes a relative hyperoxia which is further complicated by assisted ventilation, used to keep the infant alive [19, 20]. During this phase, development of retinal vessels is retracted and the endothelium is damaged secondary to hyperoxia-induced increase in free radical production and decrease in growth factor concentrations such as VEGF, Hypoxia-inducible factor 1-alpha (HIF-1α) and Insulin-like growth factor 1 (IGF-1) [21]. Angiogenesis of the peripheral avascular areas of the retina is thus incomplete during this phase [22]. Phase II, is called as hypoxic phase, which starts usually around week 32-34 and continues until the 44th gestational week [23]. During this phase, metabolic activity of retina is increased to support development of outer segments of photoreceptors in the retina, a process that requires supplemental oxygen and is therefore associated with hypoxia which, in turn, triggers the secretion of a number of angiogenic factors such as VEGF, that causes preretinal proliferation at the vascular/avascular retinal border [23]. The jack/stat signaling pathways causing an increase in free radical concentration are stimulated in the both phases [21].

Excessive production of oxidative radicals or impaired antioxidant defense systems may lead to disruption of delicate oxidant-antioxidant balance, resulting in oxidative stress and damage. Maintaining this balance in the premature infant is challenging because increased levels of oxidants associated with hypoxia, hyperoxia or inflammation cannot be counteracted by immature antioxidant defense systems. Studies consistently have shown that the antioxidant enzyme systems were improved and lipid peroxidation was reduced with increased GA [24]. One such study demonstrated that 8-hydroxy 2-deoxy guanosine level, an indicator of oxidative stress, was higher in preterm infants than term ones [25]. The risk of ROP development in premature infants is negatively correlated with GA.

CoQ10, a potent lipophilic antioxidant and membrane stabilizer involved in cell signal transduction and gene expression, is an important component of the mitochondrial respiratory chain as an electron carrier [26]. Previous studies have demonstrated the protective effects of coenzyme Q10 on various experimental disease models such as glaucoma and hypoxic neonatal rat models and neurodegenerative disorders [9-12, 27-29]. Beharry et al. [19, 29] in two recent experimental rat models of neonatal hypoxia showed that CoQ10 and n-3 polyunsaturated fatty acid supplementation reduced intermittent hypoxia-induced oxidative stress, normalized retinal layers and altered factors that influence postnatal growth. Specifically, in a rat model of oxygen-induced ROP, they showed that CoQ10 reduced VEGF, preserved astrocytic integrity, reduced neovascularization and normalized retinal layers. The authors suggested that CoQ10 and n-3 polyunsaturated fatty acid supplementation could be an alternative supportive means of ROP treatment [12, 29]. In another rat model, CoQ10 eye drops were shown to protect retinal ganglion cells from apoptosis [30].

Coqun® eye drop has recently been approved for clinical use. Fato et al. [31] in a study on patients who received Coqun® one hour before pars plana vitrectomy, provided convincing evidence that Coqun® penetrated the vitreous cavity and reached the retinal layers. Several other studies showed that Coqun® significantly reduced superoxide dismutase levels in infants with pseudoexfoliative glaucoma and also had a positive effect on infants with pattern electroretinogram (internal retinal functions) and visual evoked potential (VEP) in primary open angle glaucoma [32, 33].

Our study had some limitations, such as the retrospective design and the relatively small sample. Thus, we suggest future investigations with larger study groups and prospective design to achieve more conclusive results. We hope this study would be a prototype for further studies about supplemental anti-oxidative topical therapy in addition to conventional ROP treatment.

## CONCLUSION

In the current study, we showed that Coqun® provided faster retinal vascular maturation in infants with low-grade ROP and reduced the number of treatments in infants with APROP. This appears compatible with the biological effects of the CQ10 and Vitamin E complex which is shown to support antioxidant defense systems and suppress toxic effects of the free oxygen radicals in premature infants and thereby reduce the incidence of ROP. Given the fact that this study is limited by its small sample size and its retrospective nature, our results should be supported by further prospective multi-center studies with larger and more homogenous samples to help development of new supportive therapeutic modalities in the treatment of ROP.

## DISCLOSURE

Ethical issues have been completely observed by the authors. All named authors meet the International Committee of Medical Journal Editors (ICMJE) criteria for authorship of this manuscript, take responsibility for the integrity of the work as a whole, and have given final approval for the version to be published. No conflict of interest has been presented.

## Funding/Support:

None.
